# Tooth tissue engineering: tooth decellularization for natural scaffold

**DOI:** 10.4155/fsoa-2016-0016

**Published:** 2016-03-31

**Authors:** Luciana Aparecida de Sousa Iwamoto, Monica Talarico Duailibi, Gerson Yoshinobu Iwamoto, Yara Juliano, Michel Silvio Duailibi, Francisco André Ossamu Tanaka, Silvio Eduardo Duailibi

**Affiliations:** 1 CTCMol, Center of Cellular & Molecular Therapy, UNIFESP- Universidade Federal de Sao Paulo- Escola Paulista de Medicina, Sao Paulo, Brazil; 2 Translational Surgery, Surgery Department, UNIFESP- Universidade Federal de Sao Paulo- Escola Paulista de Medicina, Sao Paulo, Brazil; 3 National Institute of Science & Technology, Biofabrication Institute, BIOFABRIS, Campinas, São Paulo, Brazil; 4 Material Engineering, UNIFESP- Universidade Federal de São Paulo, Sao José dos Campos, Sao Paulo, Brazil; 5 Health Science Department, UNISA – Universidade de Santo Amaro, Sao Paulo, Brazil; 6 Faculdade de Ciencias Medicas Santa Casa de Sao Paulo, Sao Paulo, Brazil; 7 ESALQ- Escola Superior de Agronomia Luiz de Queiroz, Universidade de Sao Paulo, Sao Paulo, Brazil

**Keywords:** decellularization, demineralization, tissue engineering, tissue scaffold

## Abstract

**Aim:**

Tissue engineering is a multidisciplinary science that aims to produce replacement organs and biological substitutes. One of the techniques involves decellularizing a biological organ without altering its structure. One challenge is how to demonstrate which method would be better for this process.

**Methodology:**

Fifty premolar teeth were divided into five groups: G1 (control): solution of 10% formaldehyde; G2: phosphate buffer saline (PBS), 28 g of tetrasodium ethylenediaminetetraacetic (EDTA), sodium hypochlorite 2.5% (SH); G3: PBS, EDTA and 40v hydrogen peroxide (HP); G4: PBS, EDTA, SH, enzymatic detergent (ED); and G5: PBS, EDTA, HP, ED. Each group was analyzed by scanning electron microscopy (SEM), x-ray, measured weights and color and received statistical analysis.

**Conclusion:**

This study demonstrated that G5 was the most appropriate method to obtain a natural scaffold.

## Abstract (lay abstract)

**Lay abstract:** Tissue engineering is a multidisciplinary science that aims to produce replacement organs and biological substitutes. One of the keys to reproducing an organ or tissue is to find the ideal scaffold – natural or synthetic – for application in regenerative medicine. It should present a compatible macro- and micro-structure to guide the behavior of seeded cells. The objective of this work is to promote a natural scaffold by using decellularizing techniques for application in tooth tissue engineering. Fifty premolar teeth were divided into five groups, with different solutions. G5 was the best method to obtain a natural scaffold.

**Figure 1.**
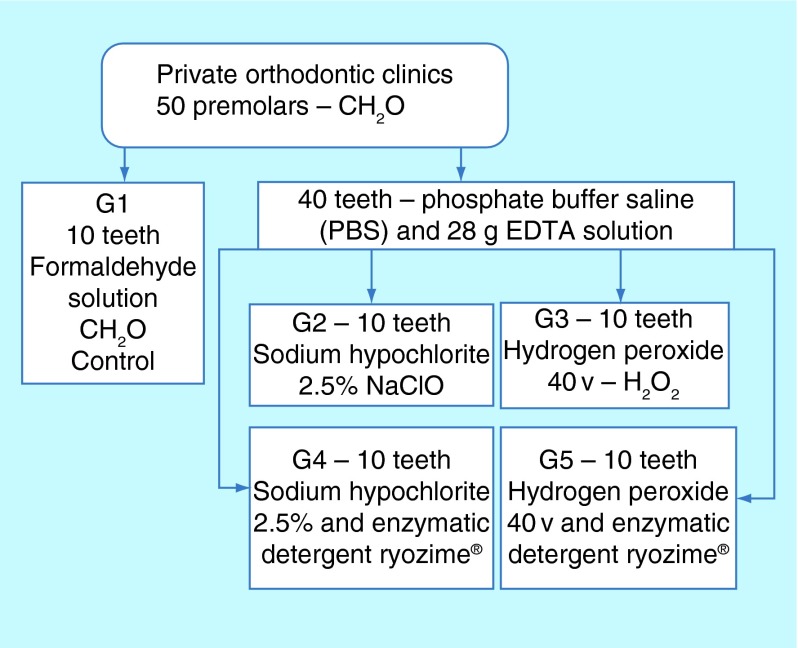
**Flowchart of method: sample distribution – 50 premolars were divided into two groups: 10 for control group and 40 for experimental groups and then, these 40 were divided in four groups of the different solutions.**

**Figure 2.**
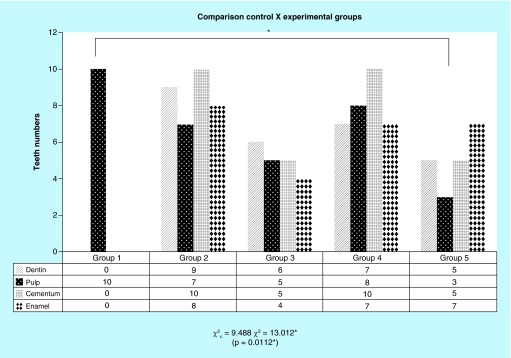
**Graph representative of microscopic structures changes on SEM. Comparisons between the experimental groups with the control group identifying significant difference on the group 5 observing all teeth structures as enamel, dentin, pulp and cementum.** The differences were measured with respect to direct comparison with the control group (group 1), presence or absence of pulp, preservation or destruction of the dentine structure, enamel and cementum, presence or absence of cracks in the cement region. *p = 0.0112.

**Figure 3.**
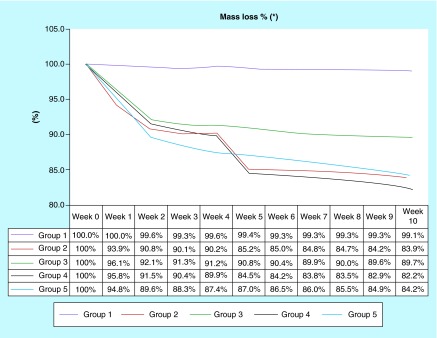
**Mass loss delta%.** All experimental groups have had mass loss during 10 weeks. On the experimental groups, the group 2 and 4 had more mass loss and both were under sodium hypochlorite solution.

**Figure 4.**
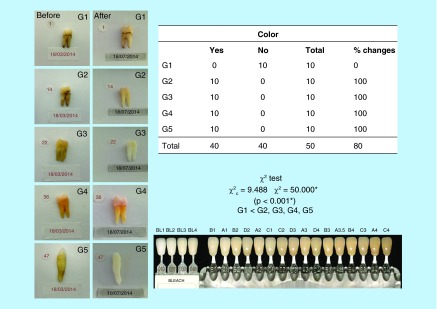
**Color analysis table.** All samples showed changes in color, with high level of significance. All experimental groups have modified their color, except the control group. Figure shows the VITA^®^ scale.

**Figure 5.**
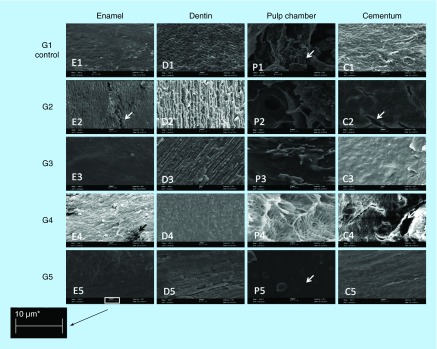
**SEM images of changes comparing structures as enamel, dentine, pulp chamber and cementum in groups (950×, 10 μm).** Analysis on SEM images from different dental tissues, enamel, pulp, cementum and dentin. G1 (control group) and then other four experimental groups. The arrows identified on pictures E2 and C2 the presence of crackers; on the picture P1 the pulp presence can be identified and on the picture P5 the pulp absence can be observed. The image revealed the absence of biological residue on the group 5 probing hydrogen peroxide and enzymatic detergent the best solution for decellularization.

The replacement of tooth loss has always been a challenge in dentistry, and the search for a biological substitute sees new therapeutic horizons. Tissue engineering (TE) is a field of study that represents the most promising approach toward organ replacement. In fact, the use of a biological substitute for restoring functional balance may be more compatible with the body than the available therapies. The principles behind TE are the existence of stem cells, tridimensional structure (scaffolds) and growth factors, resulting in the construction of a functional organ [1–5].

Scaffolds are used as a support with a macro- and micro-geometry similar to the original tissue mimicking its anatomical, functional and mechanical proprieties. This facilitates the migration and binding of transported cells or biomolecules used to replace, repair and regenerate newly formed tissue. There are two main types of scaffolds: natural and synthetic. Despite the difficulties in obtaining an organ-derived structure, this would be the optimal approach as its shape, material and design are exactly the same as the previously existing organ. Moreover, cellular material or biological residues, as cellular antigens are eliminated, are not recognized as threats by the host, thus not inciting an inflammatory response or an immune-mediated tissue rejection [3–7].

To reach it, a process named decellularization is necessary, which must not modify the organs’ structural tissue. Tissue and organ decellularizing techniques have been successfully applied in innumerous TE applications, like new biomaterials [8]. In dentistry, few evidences have been constructed in this direction. Nevertheless, the possibility exists that teeth decellularization could become a great scaffold to receive cells [9].

The most effective process of decellularization should include a combination of physical, chemical and enzymatic approaches. There are several methods used in clinical practice as they have very high variation of decellularization efficacy and time, taking from a few days to several months and structure's alteration, affecting volumetric capacity.

Enzymatic detergents have been routinely used in the process because they are effective for solubilizing collagen and removing cellular or biological residues [10,11]. Chelating agents such as EDTA cause effective cell lysis, but do not effectively remove all biological material, requiring an association with other enzymatic methods [9,12]. Formaldehyde in buffered solution, equivalent to 10% aqueous solution of formaldehyde was efficient to maintain original teeth characteristics [13]. Sodium hypochlorite (SH) is a compound used in dentistry for endodontic treatment, in order to disinfect the endodontic conduct and assists to remove the tooth pulp tissue [10,14]. Hydrogen peroxide (H_2_O_2_) is used as a bleaching agent, and when in contact with blood produces effervescent reaction, resulting in hemolysis and consequently removing all biological residues from the root canal [15].

There are many mechanisms for human tooth demineralization and decellularization, but there is not yet an evidence-based process. Since dentistry aims to achieve not only the tooth restoration but also its regeneration, there is a need for studies and protocols that biomimic the body, and result in feasible use as a natural scaffold. The aim of this work is to compare and indicate which one is the most appropriate method for decellularization and structure maintenance, applied to a 50 teeth sample. Regarding this purpose, a primary outcome was to observe the presence of pulp after undergoing experimental solutions [9].

## Materials & methods

This research was approved by UNIFESP (Universidade Federal de Sao Paulo) ethical committee under number 87191. The study was conceptualized as a primary, experimental, prospective, analytic and comparative design. In this design, 50 premolar teeth were obtained from human volunteers and collected from private clinics after orthodontic extraction procedure. Written consent was obtained for all biological tissue. Inclusion criteria for teeth selection were: healthy subjects without chronic use of any kind of drug treatment, radicular complete formation and signed written consent. Exclusion criteria: teeth sectioning for removal, dental anomaly and dental whitening.

Premolars were kept at room temperature in a Falcon tube 10% formaldehyde solution up to its transference to the laboratory, where randomly ten teeth were kept in the formaldehyde solution to be the control group (G1) and 40 were placed into a phosphate buffer saline (PBS) and 28 g tetrasodium ethylenediaminetetraacetate (EDTA) solution. After 30 days, the 40 teeth were divided into four groups and treated for 12 weeks. Groups were PBS, EDTA, SH 2.5% (SH) (G2); PBS, EDTA, 40v hydrogen peroxide (HP) (G3); PBS, EDTA, SH 2,5%, Ryozime^®^ enzymatic detergent (ED) (G4); PBS, EDTA, HP, ED (G5) (Figure 1).

Biological material was subjected to scanning electron microscopy (SEM) after 12 weeks of treatment, for the verification of dentinal tubules, root canal, enamel, dentin and pulp. With this approach, we sought to determine the presence or absence of pulp material (decellularizatiton), in order to confirm the total removal of biological residue and to check structural changes represented by demineralization of enamel, cement and dentine. The same researcher performed all scanning, predefined criteria's and determined the outcomes. These criteria were pulp existence or not; cracks and condensations at mineralized tissue (Figure 2).

The samples were weighed every 7 days throughout the study period (Figure 3). The teeth were photographed in the longitudinal direction before and after the 12-week treatment period, this record aimed to monitor macrogeometric amendments. X-ray imaging was taken with a scalimeter constructed from aluminum with 3-cm long, 1-cm wide and 1-cm height, with ten levels (0.3 cm each), and height of 0.1 cm, aiming to standardize the different shades of gray observed on x-rays.

The changes in teeth color were assessed by a visual scale VITA^®^. This is a widespread scale in dentistry and allows to objectively compare the teeth to preselected colors. Two independent observers rated color changes before and after treatments [16] (Figure 4).

## Statistical analysis

Statistical analyzes were assessed as a primary outcome by the Friedman's chi-square test comparing the presence or not of pulp or biological residues (decellularization), and alteration in dentin, enamel and cement (demineralization), after 12 weeks by scanning the image in SEM. As secondary outcomes, we analyzed perceptual teeth weight loss, across 12 weeks with the Friedman's analysis of variance. And for delta perceptual teeth weight loss before and after treatments, we used Kruskal–Wallis variance test. Color was analyzed by the χ^2^ test. The level of rejection of the null hypothesis was set at 5%.

## Results & discussion

The ‘p-value’ below 0.05 in all χ^2^ tests shows it is significative, rejecting the nullity hypothesis, meaning the results are heterogeneous. In all the cases, χ^2^_c_ = 9.488 (critical value). When the groups are compared with against G1 in terms of decellularization (pulp elimination), the pulp was reduced in all other groups, the ones treated with hydrogen peroxide (G3 and G5) showed very significant statistical advantage in comparison to groups without it (χ^2^ = 13,012; p = 0.0112). Groups with SH (G2 and G4) had the dentin highly compromised (χ^2^ = 18,196; p = 0.0011). Cracks on the cement were very affected at G2 and G4, with SH (χ^2^ = 29,167; p = 0.001). Enamel was intensively attacked at G2, G4 and G5 (χ^2^ = 17,147; p = 0.0018). Color change was highly modified in all groups, except G1 (χ^2^ = 50,000, p <0.0010) (Figure 2).

The delta percentage comparing weight lost before and after undergoing experimental solutions, showed that G2, G4 and G5 had better performance than the G1 and G3 (p < 0.0001). Moreover, the control group had insignificant loss while G3 lost approximately 10%. The percentage weight lost across 12 weeks showed the demineralization process was more intense in the first 4 weeks (Figure 3).

As to the color, all groups had significant color changes from beginning to end of treatment, except the control group G1 (p <0.001) (Figure 4).

The total sample was composed of 50 premolar teeth, divided in five groups with ten teeth each, the group G1 was selected as a control group, kept in formaldehyde to keep the original characteristics for comparative analysis with the different processes, on dentin, pulp, cement and color.

The other groups were exposed to EDTA [17] and later to different processes targeting a direct comparison between SH, hydrogen peroxide (HP), enzymatic detergent (ED) and the velocity of drying processes. They were distributed as a matrix where G2 and G4 were treated with SH, and G3 and G5 were exposed to hydrogen peroxide. Groups G4 and G5 were treated with enzymatic detergent.

It was also evaluated if the dehydration process could interfere on the result, so groups G1, G2 and G3 were submitted to fast dehydration and groups G4 and G5 to slow dehydration, the results did not evidence significant variation between the different processes.

As enamel is an extremely rigid mineral layer, to reach the internal structure is necessary to promote a demineralization step, so pulp chamber and its tissue (vessels, arteries and nerves) can be accessed. Even in the presence of chelating substance or acids, the demineralization process occurs normally, removing mineral structures from teeth and allowing sequential solutions penetrate and sanitize interior chambers. One alternative way to reach the pulp chamber could be the perforation of coronary tooth structure, but this method would compromise the extracellular matrix, turning it unfeasible to be used as a natural scaffold, reason why it was not considered in this study [18–23].

Significant variations were observed on structural properties of all groups, except group G1 (control): the reduction of calcium concentration was evidenced by the weight losses; the visual analysis exposed an obvious color variation, changes on shape, dentin and enamel (Figure 3).

SEM images exposed the difference of actuation effectiveness between the decellularization solutions, where G3 and G5 samples, using HP, kept the structural appearance on remained pulp (looked like it was corroded continually), while the G2 and G4 samples, exposed to SH, showed a damaged appearance of the pulp, looked like it was frayed [24,25].

SH is regularly used on clinical practice in endodontic irrigation and channel sanitization, which made the result quite unexpected. As the samples’ containers are transparent, it is possible the light exposition interfered in the result, as SH can have its performance reduced when exposed to sunlight (UV), although the HP also has the same restriction, and was not affected on the same level. On the other hand, HP is a powerful oxidizing agent, releasing nascent oxygen as an effervescent reaction, what can explain the higher effectiveness on decellularization at pulp chamber in groups 3 and 5.

Evidences demonstrated an intense actuation of SH in the first 4 weeks, with a performance reduction on sequence, while the same does not happen with samples exposed to hydrogen peroxide (mass losses were almost uniform during the treatment period), as demonstrated by the graphic of percentage mass losses (Figure 3).

G4, using enzymatic detergent in association to SH, presented accelerated mass reduction; a similar result could be observed in G2 (only SH). G5, also with enzymatic detergent, but with hydrogen peroxide (HP), did not react in the same way. In G2, the dentin was more affected than in the other groups (Figure 5, B2) [26–29].

Corroborating these findings, Lottanti [30] stated that the demineralizing solution associated with SH caused erosion of dentin showing it not effective yet an interesting approach. Gillette *et al*. [22] affirmed the importance of maintaining the extracellular matrix to be a differential in order to better target cell behavior.

Extracellular matrix preservation and effective removal of biological residues were considered as the main drivers, while G5 presented results significantly better than the other groups, although presenting a significant mass reduction, with 30% of samples presenting some pulp residues at the end of process. The detail visibility in this group was much cleaner, without shadows at SEM image, and with an easy identification of each component: dentin, cement, pulp and its color. This fact evidenced that the method as described did not guarantee the complete decellularization, without the occurrence of demineralization [31].

If it was considered purely demineralization, it is possible that SH could be pointed to as best performance, although a reminder must be placed here of the huge irregular behavior on mass reduction at G2 and G4, evidenced by the way SH acts, causing cracks and plates fall (Figure 3, weeks 4–5). As a matter of fact, it affects the shape and integrity of sample, showing cracks on cement (Figure 5, white arrows at C2 and C4). G3 and G5 show a more regular behavior, losing mass almost continuously, with few cracks evidenced.

Still considering mass reduction, but now analyzing enzymatic detergent (ED), it is possible to see at Figure 3 a difference between G3 (HP without ED) and G5 (HP with ED), but not between G2 (SH without ED) and G4 (SH with ED). This means ED was able to intensify the effectiveness of hydrogen peroxide, probably because of intensification of effervescence effect increasing local pressure at pulp chamber, and improving pulp removal. As an actuation form of SH is different, the same effect was not observed.

Color variation was observed in all samples, except G1, but G3 and G5 reached a higher degree on color scale (Figure 3).

X-ray images showed a significant variation on radiopacity, caused by demineralization effectiveness of EDTA [32], although shape and size changes were not evidenced – the teeth kept their macroscopic characteristics.

The dental pulp has a subpopulation of multipotential stem cells that are differentiated and better expressed when adhered to a scaffold, according to Sakai *et al*. [33]. Scaffolds that do not exhibit toxicity to the cells allow good fixation according to Mirmala *et al*. [34], and inductive materials optimize the regeneration according to Garller *et al*. [35]. Decellularization is an efficient process according to Burk *et al*. [36] and is an alternative to dentin bovine as a scaffold [37]. These findings prove that our results are approximately approaching on clinical relevance.

One of the goals of TE is to reduce the dependency of organ donation, so it is necessary to improve biomimetic models [38]; growth of our knowledge will help us to have broader evidences and to achieve the ultimate goal of dentistry related to tissue engineering, ‘the complete replacement of a lost tooth’. When possible, it will place us in a new reality, a new therapeutic treatment will be possible and the benefits for patients will be immeasurable. For sure it will lead health professionals toward unimaginable technological advances.

## Conclusion

This research demonstrated that the most appropriate method to demineralize and decellularize teeth, enabling them as natural scaffolds, was G5 (PBS, EDTA, hydrogen peroxide and Ryozime^®^ enzymatic detergent), although this presented some pulp residues in 30% of samples. Microscopic analysis demonstrated this group as the one with the least presence of biological particles, and with lower structural damage. All groups presented difference in color, except the G1. All samples submitted to SH solution showed cracks at the cement region.

## Future perspective

The perfect method to decellularize teeth was not found yet; although G5 presented better results than the others, 30% of the samples remained with biological residues. Teeth, to be feasible as natural scaffolds, must reach 100% clearance, no structural damage and have the matrix extracellular (MEC) preserved.

Improvement of the method of decellularization and sterilization for natural scaffolds, taking care about its toxicity, is required for use in ET, to seed stem cells and evaluate its final performance.

**Executive summary**

The study aimed to evaluate different methods for teeth demineralization and decellularization.
The possibility is that decellularized teeth could become a good scaffold to seed cells.
Fifty premolar teeth were divided into five groups: G1 (control), G2, G3, G4 and G5; with ten teeth each.
All groups presented difference in color, except the G1.
Sodium hypochlorite solution showed cracks at the cement region.
Hydrogen peroxide is a powerful oxidizing agent, releasing nascent oxygen as an effervescent reaction, what can explain the higher effectiveness on decellularization at pulp chamber in G3 and G5.
G5 was the most appropriate method to obtain a natural scaffold in this study.
